# Identification, Classification, and Functional Analysis of *AP2/ERF* Family Genes in the Desert Moss *Bryum argenteum*

**DOI:** 10.3390/ijms19113637

**Published:** 2018-11-19

**Authors:** Xiaoshuang Li, Bei Gao, Daoyuan Zhang, Yuqing Liang, Xiaojie Liu, Jinyi Zhao, Jianhua Zhang, Andrew J. Wood

**Affiliations:** 1 Key Laboratory of Biogeography and Bioresource in Arid Land, Xinjiang Institute of Ecology and Geography, Chinese Academy of Sciences, Urumqi 830011, China; lixs@ms.xjb.ac.cn (X.L.); liangyuqing14@mails.ucas.ac.cn (Y.L.); liuxiaojie215@mails.ucas.ac.cn (X.L.); 2 School of Life Sciences and State Key Laboratory of Agrobiotechnology, The Chinese University of Hong Kong, Hong Kong, China; gaobei@link.cuhk.edu.hk; 3 University of Chinese Academy of Sciences, Beijing 100049, China; 4 School of Life Science, University of Liverpool, Liverpool L169 3BX, UK; j.zhao46@student.liverpool.ac.cn; 5 Department of Biology, Hong Kong Baptist University, Hong Kong, China; jzhang@hkbu.edu.hk; 6 Department of Plant Biology, Southern Illinois University, Carbondale, IL 62901-6899, USA; wood@plant.siu.edu

**Keywords:** *AP2/ERF* genes, *Bryum argenteum*, transcriptome, gene expression, stress tolerance

## Abstract

*Bryum argenteum* is a desert moss which shows tolerance to the desert environment and is emerging as a good plant material for identification of stress-related genes. *AP2/ERF* transcription factor family plays important roles in plant responses to biotic and abiotic stresses. *AP2/ERF* genes have been identified and extensively studied in many plants, while they are rarely studied in moss. In the present study, we identified 83 *AP2/ERF* genes based on the comprehensive dehydrationrehydration transcriptomic atlas of *B. argenteum*. *BaAP2/ERF* genes can be classified into five families, including 11 AP2s, 43 DREBs, 26 ERFs, 1 RAV, and 2 Soloists. RNA-seq data showed that 83 *BaAP2/ERFs* exhibited elevated transcript abundances during dehydration–rehydration process. We used RT-qPCR to validate the expression profiles of 12 representative *BaAP2/ERFs* and confirmed the expression trends using RNA-seq data. Eight out of 12 BaAP2/ERFs demonstrated transactivation activities. Seven BaAP2/ERFs enhanced salt and osmotic stress tolerances of yeast. This is the first study to provide detailed information on the identification, classification, and functional analysis of the *AP2/ERFs* in *B. argenteum*. This study will lay the foundation for the further functional analysis of these genes in plants, as well as provide greater insights into the molecular mechanisms of abiotic stress tolerance of *B. argenteum*.

## 1. Introduction

*Bryum argenteum* is an important component of the desert biological soil crusts in the Gurbantunggut and Tengger Deserts of northwestern China [1,2]. *B. argenteum* has gained increasing attention as a model organism due to its comprehensive tolerances to the desert environment, such as frequent desiccation–rehydration events and high UV radiation [3,4]. Wood et al. (2007) reported that *B. argenteum* is among the most desiccation tolerant (DT) moss species and is classified as category “A” [5]. Studies on *B. argenteum* have focused on the ecological aspects of vegetative desiccation tolerance, including morphological, structural, and physiological responses to adapt to the desert environment [3,4,6,7]. *B. argenteum* is emerging as a model moss for studying the molecular mechanisms of DT and as a source of stress-related genes [8].

APETALA2/Ethylene Responsive Factor (AP2/ERF) is one of the largest transcription factor (TF) families of plants, and the family members have been demonstrated to play important roles in plant metabolism, development, and stresses response [9]. *AP2/ERF* genes have been identified and studied extensively in the context of plant stress tolerance in many plants [10,11,12]. The *AP2/ERF* gene family has been rarely studied in moss species, however, the largest TFs families found in the plant transcription factor databases (TFDB) are *AP2/ERF* genes annotated in the mosses *Physcomitrella patens* and *Sphagnum fallax* [13,14]. Moreover, *AP2/ERFs* were demonstrated to be regulated in response to multiple stresses, such as salinity and UV in *P. patens* [15], and *PpDBF1* gene was reported to confer drought, salt, and cold tolerances in transgenic tobacco [16]. Additionally, AP2/ERFs also demonstrated to be the most abundant TFs in the DT moss *Syntrichia caninervis* [17]. The majority of *DREB* (Dehydration-Responsive Element-Binding Protein) genes in *S. caninervis* responded to dehydration and/or rehydration treatments [18,19], indicating that AP2/ERF transcriptional factors also play a central regulatory role during stress responses in moss species.

AP2/ERF classification employs a well-established method based on Arabidopsis and rice [20] and this method has been widely used to classify the *AP2/ERF* family genes in many plant species [11,21]. Two classic classification methods have been proposed for the plant AP2/ERF superfamily based upon the number of AP2 domains and sequence similarities [22]. Sakuma et al. classified the AP2/ERF superfamily into five families: AP2, RAV (Related to ABI3/VP1), DREB ERF and Soloists [22]. Furthermore, *DREBs* are further classified into A1–A6 groups and *ERFs* are divided into the groups B1–B6 [22]. Nakano et al. (2006) classified AP2/ERF proteins into three major families: AP2, ERF (include both DREBs and ERFs) and RAV [20]. The ERF family is then further sub-divided into twelve groups in Arabidopsis and fifteen groups in rice according to the structure and similarity of the AP2 domain [20].

High-throughput sequencing has been an effective tool to identify stress-related genes, and transcriptome-based identification and selection of *AP2/ERF* superfamily genes have been widely used in many non-model plants, such as *Hevea brasiliensis* [23], tea [24], as well as the desiccation tolerant moss *S. caninervis* [19]. Previously, we generated a de novo transcriptome for *B. argenteum* and established a desiccation–rehydration transcriptomic atlas which covered five different hydration stages [8,25]. Using this combined dataset, we established a fully comprehensive desiccation–rehydration transcriptome dataset containing 76,206 high-quality *B. argenteum* transcripts. We found that AP2/ERF was the second largest TF family in *B. argentum*. Moreover, *AP2/ERF* genes were also the most abundant differentially expressed TFs during the desiccation and rehydration process (Figure S1), indicating that the *AP2/ERF* family genes play key roles in *B. argentum* response to moss specific desiccation–rehydration process. Hence, in this study, we aimed to identify and classify the *AP2/ERF* gene family in *B. argentum* based on a comprehensive transcriptome dataset. Additionally, we investigated the gene expression patterns of *BaAP2/ERF* genes under desiccation–rehydration treatment based on RNA-seq data as well as real-time quantitative PCR (RT-qPCR) assay, and their transactivation activities were also analyzed. We further evaluated the stress tolerance ability of eight representative *BaAP2/ERF* genes in yeast system. This is the first report on identification, classification, characterization, and evaluation the stress tolerance functions of the *AP2/ERF* gene family in desiccation tolerant moss *B. argentums*. This study will provide candidate genes for molecular breeding to improve crop stress tolerance, and could be helpful for understanding the molecular mechanisms of the stress responses in *B. argentum*.

## 2. Results

### 2.1. Identification of the AP2/ERF Family Genes in B. argenteum

Based on Hidden Markov model (HMM) profiles and BLAST search, 83 AP2/ERF predicted proteins were identified from the *B. argenteum* transcriptome. The unigenes ranged from 559 to 8076 bp in length, and the corresponding deduced polypeptide sequences ranged from 145 to 1983 aa. The sequences of 83 *AP2/ERFs* were submitted to Genbank under accession numbers MK170284-MK170366. Among these 83 unigenes, 73 genes have an intact open reading frame (ORF) (87%), ranging from 163 to 1983 aa. AP2 domain analysis demonstrated that all 83 predicted proteins had full-length AP2 domains (ca. 60 aa) (100%). Seven out of 83 genes had two AP2 domains, one gene had both an AP2 and a B3 domain, while the other 75 genes had a single AP2 domain. Based on the number of AP2 domains, BaAP2/ERFs can be preliminarily classified as follows: 7 genes having two AP2 domains were classified as members of the AP2 family, 1 gene with both an AP2 and B3 domain was classified as a member of RAV family, and the 75 remaining genes with only one AP2 domain were considered as ERF family members (including both DREBs and ERFs).

### 2.2. Classification of the AP2/ERF Genes in B. argenteum

**Figure 1:**
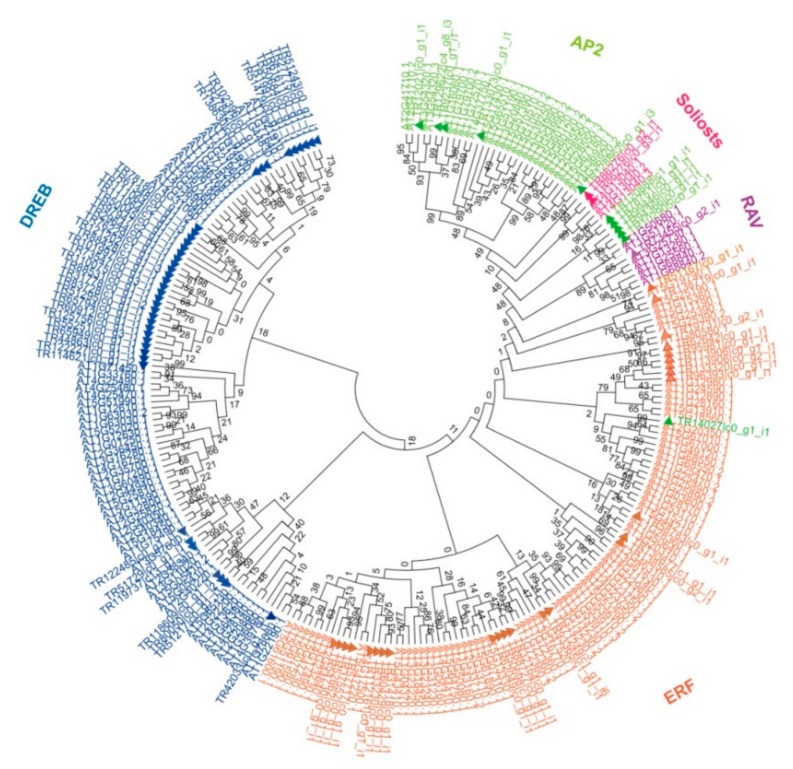
Phylogenetic analysis of AP2/ERF superfamily genes in *B. argenteum* and Arabidopsis. The gene tree was constructed using the neighbor-joining method using 83 BaAP2/ERFs and 176 AtAP2/ERFs, the evolutionary distances were computed using the Poisson correction method with pairwise deletion. Bootstrap values from 1000 replicates were used to assess the robustness of the tree. Different subfamilies were marked with various colors, the BaAP2/ERFs were labeled with rectangles to distinguish from AtAP2/ERF.

Family classification was further confirmed by constructing the phylogenetic tree using the AP2 domain of AP2/ERF in *B. argenteum*, the model plant Arabidopsis and the model moss *P. patens*. We first generated the gene tree of AP2/ERFs with *B. argenteum* and Arabidopsis. The tree showed that *BaAP2/ERF* genes were divided into five families according to Arabidopsis’ classification method, including AP2, DREB, ERF, RAV and Soloist (Figure 1). Almost half of the *B. argenteum BaAP2/ERF* genes grouped into the DREB family (with 43 members). The second largest group was the ERF family (27 genes), followed by the AP2 family (10 genes), RAV (1 gene) and Soloists (2 genes). However, the number of the AP2 family genes classified by gene tree was inconsistent with the classification result based on the AP2 domain counting. There were two inconsistencies: one was the TR14027|c0_g1_i1 gene which had two AP2 domains, therefore should be classified as AP2 family gene, while it was grouped together with ERF family in the gene tree, and the other was five genes had single AP2 domain but were clustered together with AP2 family in Arabidopsis. To further confirm their classification, we generated another two gene trees using only *BaAP2/ERF* genes (Figure S2), and with *AP2/ERF* genes both in *B. argenteum* and the model moss *P. patens* (Figure S3). We conclude that there were 11 AP2s, 43 DREBs, 26 ERFs, 1 RAV and 2 Soloists in *B. argenteum* based on the available transcriptome data.

**Figure 2:**
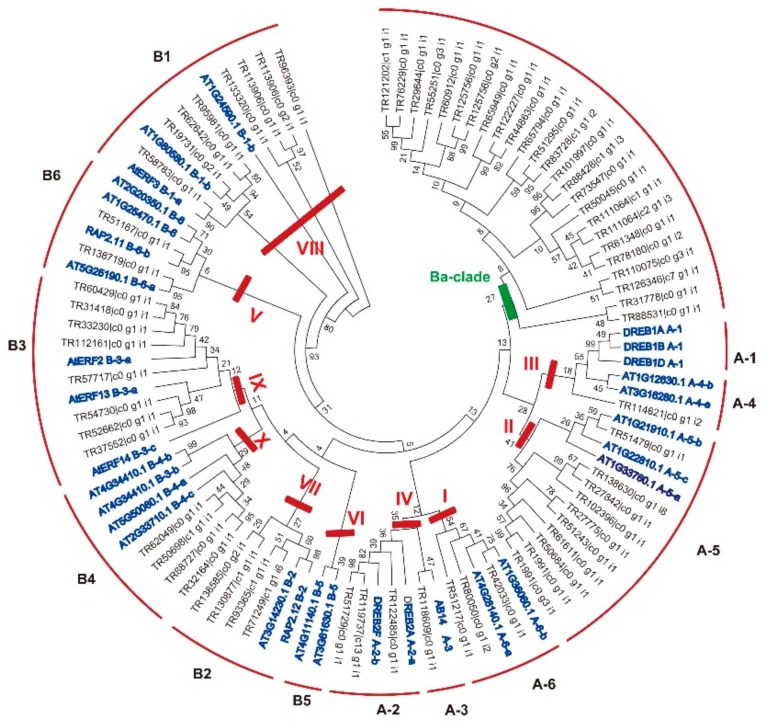
Phylogenetic analysis of ERF family genes in *B. argenteum* and Arabidopsis. The gene tree was constructed using 69 BaERFs and 31 AtERFs representative of each subfamily or group of ERF family genes in Arabidopsis. The evolutionary distances were computed using the neighbor-joining method and Poisson model with pairwise deletion. Bootstrap values from 1000 replicates were used to assess the robustness of the tree. To distinguish ERFs from *B. argenteum* and Arabidopsis, AtERFs and BaERFs were marked in blue and dark, respectively. Previously reported subfamily names (A1–A6 and B1–B6) and group names (Group I to Xb–L) were employed [20,22]. The Bryum-unique clade (Ba-unique) was labeled in green, and other groups were labeled in red.

DREB and ERF are the two major families in the plant AP2/ERF superfamily, and which are demonstrated to play important roles in abiotic and biotic stress response. DREB and ERF can be subdivided into twelve subfamilies, namely A1–A6 and B1–B6 [22], or ten groups named Groups I–X [20], and each group/subfamily have different functions [11]. To detail classify the *DREB* and *ERF* family genes, we constructed a phylogenetic tree using 69 BaERFs (including 43 DREBs and 26 ERFs) and 31 ERFs in Arabidopsis representative of each subfamily/group. We identified 10 out of 12 DREB/ERF subfamilies in *B. argenteum* based upon the classification method of Sakamu et al. [22], A2 (3 genes), A3 (1 gene), A4 (1 gene), A5 (10 genes), A6 (3 genes), B1 (8 genes), B2 (4 genes), B3 (8 genes), B4 (4 genes), and B6 (2 genes), while no members of the A1 and B5 subfamilies were found in BaAP2/ERFs (Figure 2). Accordingly, based on Nakano et al.’s classification method [20], *BaAP2/ERF* superfamily genes contained 9 out of 10 groups (lacking Group VI), including Groups I–X, and the members for each group were Group I (3 members), Group II (10 members), Group III (one member), Group IV (4 members), Group V (2 members), Group VII (4 members), Group VIII (8 members), Group IX (8 members) and Group X (4 members) (Figure 2). Moreover, we found that 26 *ERFs* can be clearly classified into specific subfamilies/groups, while more than half of *DREBs* (25/43 genes) cannot be classified to any exist subfamilies, which was clustered into one separate clade and was named as *Bryum* group (Ba-clade) (Figure 2). To know whether these Ba-clade genes are specific to Bryum or moss species, we first performed BLASTp search in NCBI database using the full-length sequence of 25 Ba-clade DREBs/ERF and found that all 25 Ba-clade DREBs have homologies in other plants, with the highest sequence identities to model moss *P. patens*. We then performed BLASTp search in ONE KP database with mosses, liverworts, and hornworts transcriptome data. The result also show that all Ba-clade genes have homologies in mosses, liverworts, and hornworts. Moreover, half of Ba-clade DREBs shared highest sequence identities to *Funaria* (Table S1).

### 2.3. Conserved Amino Acids and Motifs Analysis of BaERF Genes

**Figure 3:**
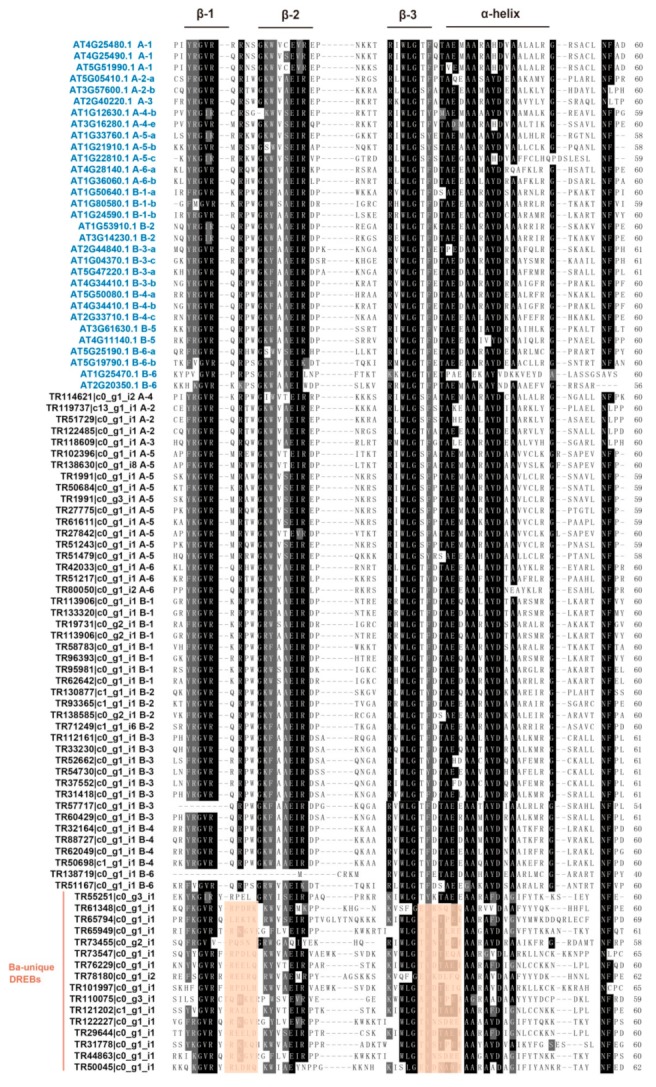
Sequence alignments of AP2 domains of representative ERF proteins in *B. argenteum* and Arabidopsis. Thirty-one AtERF genes representative of each ERF subfamily/group were aligned with 69 BaERFs. The subfamily for each ERF is depicted on the right. The locus names of AtERFs and BaERFs are marked in blue and black, respectively. The identical and conserved amino acid residues are indicated with black and light gray shading, respectively. The black bars represent three β sheets and α helix regions. The Ba-unique DREBs are grouped in pink bar, and two regions representing diverse amino acids compositions are marked with pink boxes.

**Figure 4:**
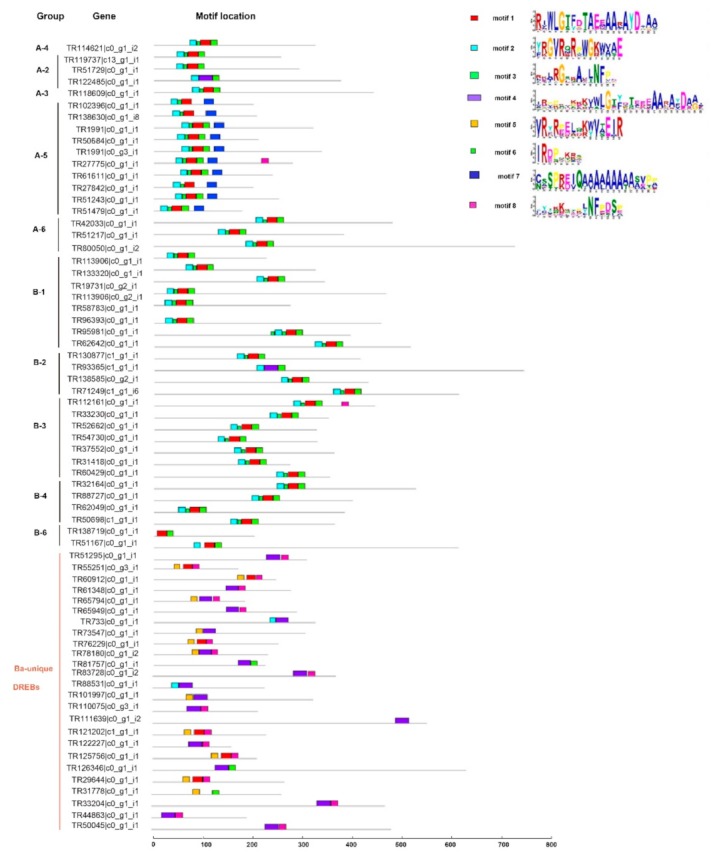
Motif analyses of BaERFs with intact ORFs using MEME online software. BaAP2/ERF proteins with complete ORFs were used for motif prediction. Parameters are as follows: any number of repetitions per sequence, motif width ranges of 6–50 amino acids, and 8 as the maximum number of motifs. Each of the sequence has an E-value less than 10. Motif composition and deduced amino acid sequence of each motif are presented.

To analyze the amino acids conservation of the AP2 domains, 69 BaERF deduced polypeptide sequences were aligned with 31 AtERFs representative of each gene subfamily in Arabidopsis. Multiple sequence alignment showed that BaERF sequences shared significant amino acid similarity with AtERFs except Ba-clade DREBs (Figure 3). Ba-clade DREBs did not belong to either subfamily/group based on existing classification. Ba-clade DREBs had more diverse amino acids composition in the AP2 domain, especially in the region between two β-sheets and the α-helix (marked with pink boxes). For example, a consensus sequence “TAE” in the C-terminal of α-helix was very conserved among AtERFs and BaERFs, however, in Ba-clade DREBs, TPE and TEE/Q/I patterns also existed (Figure 3). The motif composition analyses also supported this phenomenon. Eight motifs were detected in 69 BaERFs in total; among them, motifs 1–3 represented the typical AP2 domains in Arabidopsis as well as most of the well-classified BaERFs, of which motif 1 contained the β3 sheet and α-helix of AP2 domain, motif 2 corresponded to β1 and β2 sheet, and motif 3 was located in the very C-terminal of the AP2 domain (Figure 4). Motif 4 was similar to motif 1 which corresponded to the β3 sheet and α-helix; in the same way, motif 5 was similar to motif 2 which represent β1 and β2 sheet, and motif 8 was similar to motif 3, but their amino acids compositions differed from the classic AP2 domains in Arabidopsis. Interestingly, motif 4, 5 and 8 constituted the unique Ba-clade DREB AP2 domain. Additionally, we identified motif 7 as an A-5 DREB specific amino acids pattern.

### 2.4. Gene Expression Analysis of all the BaAP2/ERFs during Moss Specific Dehydration–Rehydration Process Using RNA-seq Data

**Figure 5:**
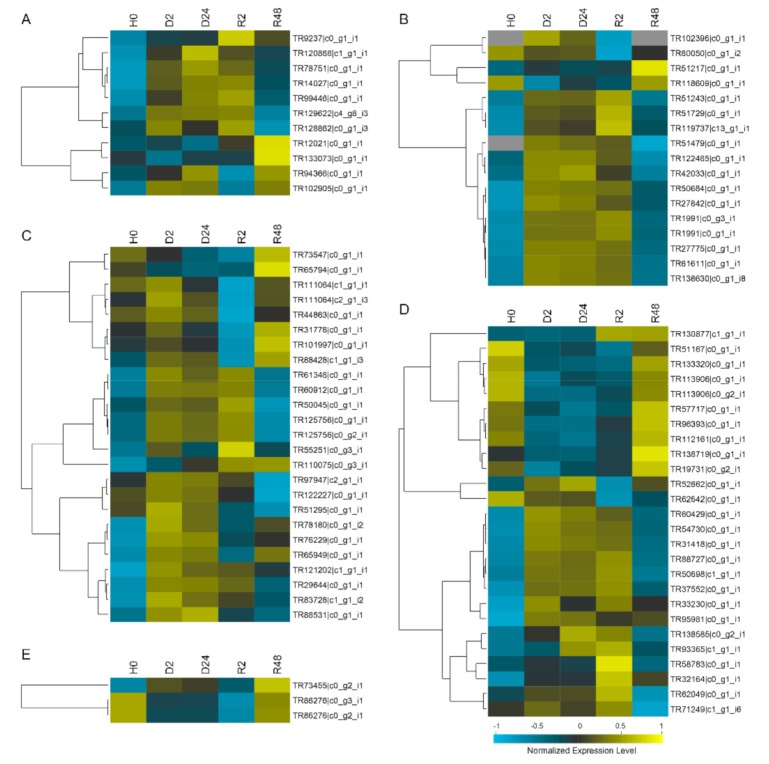
Heat map of the relative expression levels of all identified BaAP2/EFR genes during dehydration–rehydration process of *B. argenteum*. Color scores were normalized by the log2 transformed counts of RPKM values. Yellow represents high expression, while blue represents low expression. Expression differences in the transcripts were clustered by the hierarchical complete linkage clustering method using an uncentered correlation similarity matrix. Prior to the clustering analysis, expression data in unit of FPKM were pretreated using the standardization tools in Cluster 3.0. The heat maps were drawn using the Java Treeview package. Expression profiles (in log2 based values) of the: (**A**) *AP2*; (**B**) *DREB*; (**C**) unclassified *DREB* group (Ba-unique clade); (**D**) *ERF*; and (**E**) *RAV* and *Soloist* genes in *B. argenteum* response to dehydration–rehydration treatment.

To evaluate the potential function of 83 *BaAP2/ERFs* genes under dehydration–rehydration stress treatment, we investigated the gene expression pattern based on the RNA-seq datasets (H0, D2, D24, R2, and R48). The results show that all 83 *BaAP2/ERFs* belonged to AP2, DREB, ERF, RAV and Soloists families exhibited elevated transcript amounts during dehydration–rehydration process (Figure 5). The majority of *BaAP2/ERF* transcripts were more abundant in both dehydration (D2 and D24) and early-rehydration (R2) stages. For example, 6 out of 11 *AP2* family transcripts and 13 out of 17 *DREB* transcripts were more abundant in dehydration–rehydration stages. Some transcripts also showed different expression patterns of accumulation. TR88531|c0_g1_i1 and TR76229|c0_g1_i1 (Bryum-unique *DREB* genes) transcripts were more abundant in D2 and D24, while TR110575|c0_g3_i1 (Bryum-unique *DREB* gene) and TR130877|c0_g1_i1 (*ERF* gene) were more abundant in R2 and R48. 

### 2.5. Diverse Gene Expression Patterns of Twelve BaAP2/ERFs during Dehydration–Rehydration Process Using RT-qPCR

**Figure 6:**
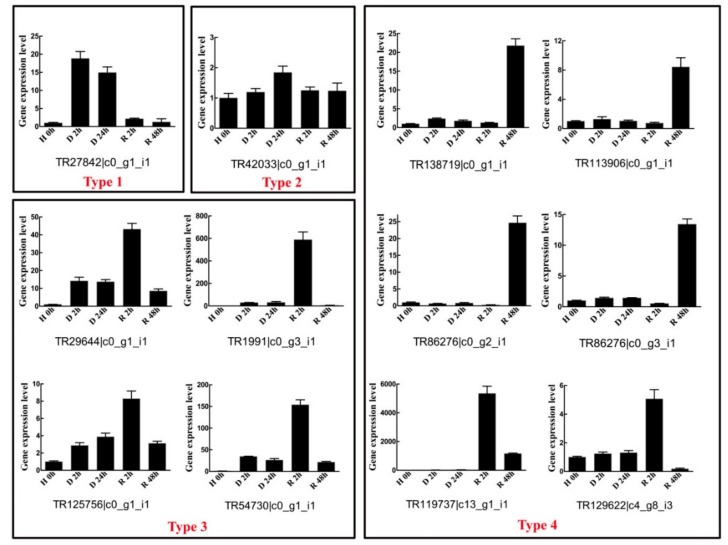
RT-qPCR validation of gene expression patterns of 12 representative *BaAP2/ERF* genes during *B. argenteum* dehydration–rehydration process. RT-qPCR quantitative gene expression data are shown as the mean ± SE. The relative gene expression levels were calculated relative to 0 h and using the 2^−ΔΔ*C*t^ method.

The expression profiles of 12 transcripts, representative of different family members of *BaAP2/ERF* (one *AP2*, four *DREBs*, two Ba-unique *DREBs*, three *ERFs* and two *Soloists*) that demonstrated a diverse pattern of induced gene expression were validated by RT-qPCR (Figure 6). RT-qPCR results confirmed the expression trends observed with RNA-seq data. RT-qPCR demonstrated that 9 out of 12 genes increased, reached a peak and then decreased, while the other three genes changed slightly and rapidly increased to the maximum fold at rehydration (R48) stage. Most genes reached an expression peak at rehydration stage (R2 or R48), except TR27842|c0_g1_i1 and TR42033|c0_g1_i1, which peaked at D2 and D24, respectively (Figure 6). The expression pattern can be divided into four types: (1) transcripts that accumulate in response to desiccation stress (e.g., TR27842|c0_g1_i1 which was strongly induced by desiccation treatment (almost 20-fold compared to H0) and rapidly reduced after rehydration); (2) transcripts which modestly accumulate in response to both desiccation and rehydration (e.g.,TR42033|c0_g1_i1 gene, the gene expression level of which changed within two-fold during desiccation and rehydration process); (3) transcripts which accumulate in response to desiccation and remain elevated upon rehydration (e.g., TR29644|c0_g1_i1, which was 10-fold increased after desiccation treatment, and then reached a peak (more than 40-fold) at R2 stage); and (4) transcripts which accumulate in response to rehydration (e.g., TR138719|c0_g1_i1, which was highly induced by rehydration (R48)).

### 2.6. Transactivation Activity Analyses of Twelve BaAP2/ERFs

**Figure 7:**
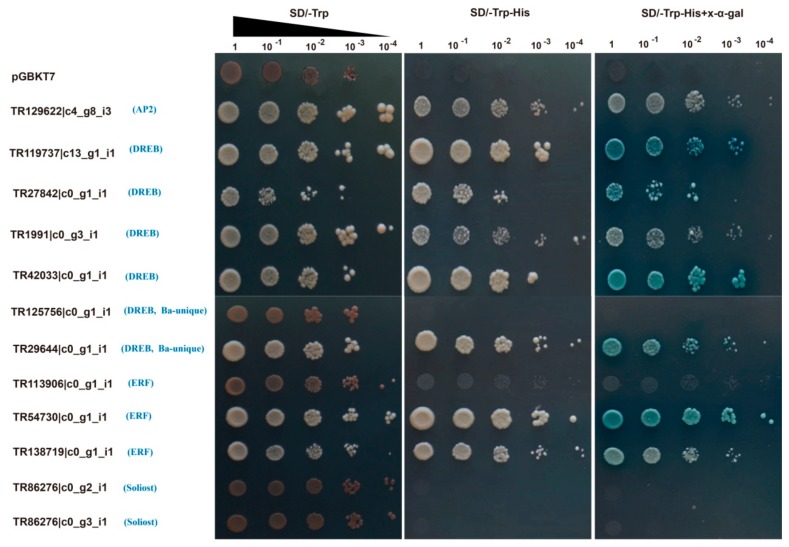
Transactivation activities of 12 BaAP2/ERF proteins in yeast. Yeast cells Y2H expressing the fusion proteins were cultured and adjusted to an OD600 of 2.0, then series diluted and dropped with 2 μL on nutritional selective medium SD/−Trp, SD/−Trp−His and SD/−Trp−His+x-α-gal. Yeast cells expressing the empty vector pGBKT7 was used as negative control. Photos were taken after incubating at 30 °C for 2–4 days.

We further investigated the transactivation activity of the above 12 *BaAP2/ERFs* using a yeast-based transcriptional activity assay. The results show that 8 out of 12 BaAP2/ERFs proteins can grow well on SD-Trp, SD-Trp-His medium and exhibit α-galactosidase activity on SD-Trp-His medium containing x-α-gal (Figure 7). This indicates that these AP2/ERF proteins (one AP2 (TR129622|c4_g8_i3), four DREBs (TR119737|c13_g1_i1, TR27842|c0_g1_i1, TR1991|c0_g3_i1, and TR42033|c0_g1_i1), one Ba-unique DREB (TR29644|c0_g1_i1) and two ERFs (TR54730|c0_g1_i1 and TR138719|c0_g1_i1)) demonstrate transactivation activities. Four proteins (one Ba-unique DREB (TR125756|c0_g1_i1), one ERF (TR113906|c0_g1_i1) and two Soloists (TR86276|c0_g2_i1 and TR86276|c0_g3_i1)) grew similarly to the negative control indicating that these proteins might not function as transcriptional activators in this yeast heterologous system.

### 2.7. Stress Tolerance Ability Evaluation in Transgenic Yeast

**Figure 8:**
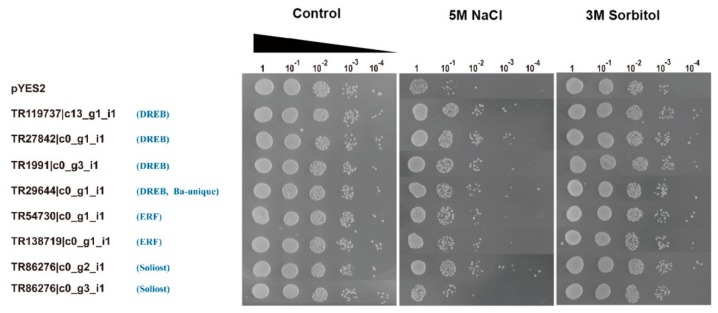
Growth of *S. cerevisiae* yeast cells transformed with the pYES2-BaAP2/ERFs under salt and osmotic stress conditions. To test the salt and osmotic tolerances, the same quantity of yeast culture sample was re-suspended in 5 M NaCl and 3 M Sorbitol, respectively, at 30 °C for 45 h. For non-stress control, an equivalent number of yeast cells was re-suspended in 200 μL of sterile water and incubated at 30 °C for 45 h. Serial dilutions of 1:10 transformed yeast cells were grown on SC-ura medium for two days.

To investigate the ability of BaAP2/ERF proteins to enhance abiotic stress tolerance in heterologous expression system, eight representative *BaAP2/ERFs*, driven by a galactose-inducible promoter (pYES2), were introduced into *S. cerevisiae* (INVSc1). After 45 h of exposure to 5 M NaCl and 3 M Sorbitol, the growth patterns of all BaAP2/ERF transformed *S. cerevisiae* (pYES2-*BaAP2/ERF*) were similar to the empty vector (pYES2) under non-stress conditions (Figure 8). Seven out of eight *BaAP2/ERF* (except TR86276|c0_g3_i1) transformed *S. cerevisiae* survived better than the empty vector under salt and osmotic stresses, especially under salt stress, indicating that these seven BaAP2/ERF proteins (TR119737|c13_g1_i1, TR27842|c0_g1_i1, TR1991|c0_g3_i1, TR29644|c0_g1_i1, TR54730|c0_g1_i1, TR138719|c0_g1_i1, and TR86276|c0_g2_i1) were functional in yeast cells and improved the yeast tolerance to salt and osmotic stresses.

## 3. Discussion

*AP2/ERF* genes play central roles during plant stress responses and have been widely identified in both dicotyledonous and monocotyledonous plants using genomic or transcriptomic data [20,22], however, little is known about the functions of *AP2/ERF* genes in moss species. Several recent studies have demonstrated that AP2/ERF transcription factors play an important role in the stress responses of bryophytes [15,16,19]. *B. argenteum* is extremely tolerant to desiccation stress and is a promising model for the identification of stress related genes [8], however, no *AP2/ERF* gene in *B. argenteum* has been reported until now.

The AP2/ERF family of TFs in Arabidopsis comprises five subfamilies of TFs, classified based on sequence similarity, number of AP2 domains, and the presence of other characteristic domains [26]. It is reported that different gene families have different functions. The *AP2* gene family is associated with plant flower development, while *ERF* and *DREB* family genes accumulate in response to biotic and abiotic stress, respectively [9]. Genes annotated to a specific group within the same gene family are also reported to have different functions. A-1 type *DREB* transcripts accumulate in response to cold stress, while A-2 type *DREB* transcripts accumulate in response to osmotic and heat stresses [27]. Hence, a precise and detailed classification of *AP2/ERF* genes within a genome is an important tool for predicting gene expression and function.

Classification of the AP2/ERF superfamily is based on the number of AP2 domains and by constructing a phylogenetic tree comparing the moss AP2 domains to Arabidopsis or rice. The resulting gene tree construction has been a classic and reliable method of annotation which is widely employed in many non-model plants [24,28,29,30,31]. However, classification by gene tree should be used cautiously as the results can be inconsistent with an AP2 domain counting based classification. *Soloist* genes with a single AP2 domain always grouped together with *AP2* family genes which have two domains (as reported in *Hevea brasiliensis* and *Vitis vinifera* [23,28]). In this study, we found that *BaSoloists* clustered with *AtSoloists* and were mixed together with *AP2* family genes. In addition, five genes which contained a single AP2 domain also clustered with *AP2* family genes in Arabidopsis. To confirm the classification as AP2 family members, we constructed two more phylogenetic trees: one using only *AP2/ERF* genes in *B. argenteum* and another one using *AP2/ERF* genes from the model moss *P. patens*. Finally, these five genes were classified into *AP2* family given their greater homology with the *AP2* family genes.

The AP2 domain of *AP2/ERF* genes is conserved in plants, however amino acid variations within the AP2 domain have been documented and can refine classification of the gene [23,29]. For example, the motifs “HLG” and “WLG” in the β3 sheet can distinguish a *Soloist* gene from an *ERF* gene. Li et al. (2017) demonstrated the “EVR” motif pattern was only present in the A-1 group of *DREB* genes and “ERK” was specific to the B-6 subfamily of *ERF* genes in the β2 sheet of AP2 domain [19]. Based on this A-1 DREB-specific amino acid, in the present study, we finally confirmed the TR11462|c0_g1_i2 gene was A-4 type of DREB rather than A-1 type. Specific motif elements are also helpful for robust gene classification. In the DREB family, ERF-associated amphiphilic repression (EAR) motif was specifically present in the A-5a group genes, which contained (L/F) DLN (L/F) xP residues and may be essential for repression function [32,33,34].

Moss species have unique genes which are challenging to annotate compared to other organisms [19]. In *S. caninervis*, the majority of *ScERF* genes can be classified while few *ScERF* genes are not clustered with any Arabidopsis group, and clustered as a unique clade [19]. Similarly, in this study, half of *DREBs* cannot be classified relative to other plant genes and clustered as a Ba-unique clade. The amino acids compositions of AP2 domains were also supported that Ba-unique clade genes have diverse amino acids composition and showed more diverse motif patterns compared with other *DREB* genes. Furthermore, BLASTp search in One KP and NCBI database showed that, although Ba-unique *DREBs* have homolog genes in angiosperm, they shared very low amino acid identities. Some Ba-unique *DREBs* had very high sequence identities with other moss genes, while these moss genes were rarely characterized and no functional analysis were reported until now. Our results extend the idea that *AP2/ERF* genes in moss species can be different from angiosperm genes, and moss-unique genes may have novel and/or altered functions. It is necessary to explore their functions in future work.

Gene expression pattern was considered to be directly connected with the gene function [35], and, in this study, the expression of all 83 *BaAP2/ERFs* genes were induced during dehydration–rehydration process. Moreover, the 12 representative *BaAP2/ERF* genes in different families exhibited differential expression in response to dehydration and rehydration treatment. Within the same family, the gene expression patterns were different suggesting a functional diversity of *AP2/ERF* genes in response to dehydration and rehydration stress in *B. argenteum*. AP2/ERF proteins are important transcriptional factors which can activate many down-stream genes, thus improving the overall stress tolerance of plants [10]. In the present study, 8 out of 12 BaAP2/ERFs proteins demonstrated transactivation activity in the yeast system. Based upon patterns of gene expression and transactivation activity analysis, we selected eight representative *BaAP2/ERF* genes for further functional test in yeast and the result showed that seven of them improved the yeast tolerance to salt and osmotic stresses. Our results demonstrated that *BaAP2/ERFs* genes play crucial roles in *B. argenteum* response to stresses.

## 4. Materials and Methods

### 4.1. Identification of the AP2/ERF Protein Family in B. argenteum

*B. argenteum* transcripts (76,206) were obtained from a hydration–dehydration–rehydration transcriptome [25] (data were deposited at NCBI-SRA with accession SRP077772, https://www.ncbi.nlm.nih.gov/sra/?term=SRP077772) and served as the source for the *AP2/ERF* gene identification and presented in this study. Two methods were used together to identify the putative *AP2/ERF* genes from *B. argenteum.* Firstly, 176 Arabidopsis AP2 predicted amino acid sequences and 171 *P. patens* AP2 predicted amino acid sequences were downloaded from the plant transcription factor database (PlantTFDB v3.0) (http://planttfdb.cbi.edu.cn/) [14], and used as queries to search against the *B. argenteum* transcriptome database using tBLASTn program (E value of 1 × 10^−3^. Second, the HMM profiles PF00847 (AP2 domain) and PF02362 (B3 domain) were downloaded from Pfam database v27.0 (http://pfam.sanger.ac.uk/) [36], and the profiles were queried using hmm search command included in the HMMER (v3.0) software (E value cutoff at 1 × 10^−3^). All candidate *BaAP2/ERF* genes identified through these two methods were confirmed with Conserved Domain Database (CDD http://www.ncbi.nlm.nih.gov/cdd/) [37] and SMART (http://smart.embl-heidelberg.de/) [38] searches to ensure the presence of an AP2 domain. An AP2 domain, length of approximately 60 amino acids was considered to be a full-length AP2 domain [23]. All the predicted peptide sequences were filtered with a minimum length of 80 amino acids. Sequences which shared >98% matches were considered redundant.

### 4.2. Sequence Analysis and Classification of BaAP2/ERF Genes Using Phylogenetic Tree 

ORFs were predicted with the ORF Finder at NCBI (http://www.ncbi.nlm.nih.gov/gorf/gorf.html). Protein sequence motif detection was performed with MEME program (http://meme-suite.org/index.html) [39] using the parameters: zero or one repetition per sequence, motif width ranges of 6–40 amino acids, and 8 as the maximum number of motifs. Multiple sequence alignment was performed with ClustalW [40], phylogenetic trees were constructed by the neighbor-joining method (with 1000 bootstrap replicates) using MEGA 6.06 the evolutionary distances were computed using the Poisson correction method with pairwise deletion. Sequence similarity was analyzed using BLASTp search with NCBI and ONE KP (https://db.cngb.org/onekp/) database. All the *BaAP2/ERF* sequences were submitted to the GenBank database using *Bank*It (http://www.ncbi.nlm.nih.gov/BankIt/).

### 4.3. Gene Expression Analysis of BaAP2/ERF Genes Using RNA-seq Data

Expression differences in the transcripts under dehydration–rehydration condition were clustered by the hierarchical complete linkage clustering method using an uncentered correlation similarity matrix. Prior to the clustering analysis, expression data in unit of Fragments Per Kilobase of transcript per Million fragments mapped (FPKM) were pretreated using the standardization tools in Cluster 3.0: (a) log transform data; (b) center genes (mean); and (c) normalize genes [41]. The heat maps were drawn by using the Java Treeview package [42,43].

### 4.4. Gene Expression Pattern Analysis of BaAP2/ERF Genes Using RT-qPCR Assay

*B. argenteum* gametophytes were cultured in solid Knop medium at 25 °C with 16 h/8 h photoperiod in a climate chamber as described previously [8]. For desiccation–rehydration treatment, the well-hydrated gametophytes in Knop solid medium were transferred to 90 cm open Petri dish and air-dried for 2 h (D2) and 24 h (D24), and the desiccated gametophytes (D24) samples were subsequently rehydrated with deionized water for 2 h (R2) and 48 h (R48). All treatments were performed at 25 °C with RH ≈ 25–27%, and the well-hydrated gametophores in Knop medium without any treatment was served as the control (H0). Three biological replicates were collected for each of the time point of different treatments.

Total RNAs of *B. argenteum* gametophytes were extracted using MiniBEST plant RNA kit (Takara, Japan). Gel electrophoresis and a NanoDrop 2000 spectrophotometer (Thermo Fisher Scientific, Waltham, MA, USA) were used for RNA quality test and quantitative analysis. High quality RNA samples were used for subsequent reverse transcription. First strand cDNA was synthesized using PrimeScript^TM^ RT reagent kit (Takara, Shiga Prefecture, Japan). 

Twelve *BaAP2/ERF* genes representative different groups/subfamilies were selected to verify the gene expression pattern obtained from transcriptome data under desiccation and rehydration condition. RT-qPCR primers were designed with Primer Premier 5.0 and the primer specificities were tested by running BLAST search against the local *B. argenteum* transcriptional data. Each primer pair was further assessed using melting-curve analysis after RT-qPCR. All primer information for RT-qPCR is shown in Table S2. RT-qPCR experiments were carried out using CFX96 Real-Time PCR Detection System (Bio-Rad, Hercules, CA, USA) with SYBR *Premix Ex Taq*^TM^ kit (Takara, Shiga Prefecture, Japan). The PCR reaction mixture consisted of 2 μL cDNA sample (1:5 diluted), 0.4 μL each of the forward and reverse primers (10 μM), 10 μL master mix and 7.2 μL PCR-grade water in a final volume of 20 μL. Three biological replicates and three technical replicates of each biological replicate were used for all samples. The RT-qPCR program was as follows: initial denaturation step of 30 s at 95 °C and 40 cycles of PCR (94 °C for 5 s and 60–62 °C for 30 s). The gene relative expression levels were calculated relative to the H0 samples using the 2^−ΔΔ*C*t^ method. The *ACT* gene was used to normalize the RT-qPCR data [8]. Figures were generated using Sigmaplot 12.0.

### 4.5. Gene Cloning, Vector Construction and Transcriptional Activation Analysis in Yeast Cells

To further evaluation of transactivation activity of 12 *BaAP2/ERF* genes, we cloned these 12 genes into the pMD18-T clone vector. After sequence analysis, the PCR products of these genes were cloned separately into the pGBKT7 vector using the in-fusion PCR cloning system (Clontech, Mountain View, CA, USA). Positive plasmids containing different *BaAP2/ERF* genes were transformed into the Y2H yeast strain (Clontech, Mountain View, CA, USA). All primers used for cloning and vector construction are listed in Tables S3 and S4. The cell concentration of yeast positive transformants were adjusted to an OD600 of 2.0, the yeast cells were then diluted serially (1, 10^−1^, 10^−2^, 10^−3^, and 10^−4^) and dropped with 2 μL on synthetic dropout (SD) medium without tryptophan (SD/−Trp), without tryptophan and histidine (SD/−Trp−His), and with SD/−Trp−His plates containing x-α-gal with the final concentration of 40 mg/L (SD/−Trp−His+x-α-gal). Yeast cells expressing the empty vector pGBKT7 was used as negative control. The plates were incubated at 30 °C for 2–4 days before photographing. Adobe Illustrator CS5 was used for image processing.

### 4.6. Stress Tolerance Studies in Yeast

Eight representative *BaAP2/ERF* genes including four *DREBs* (TR119737|c13_g1_i1, TR27842|c0_g1_i1, TR1991|c0_g3_i1 and TR29644|c0_g1_i1), two *ERFs* (TR54730|c0_g1_i1 and TR138719|c0_g1_i1) and two *Soloists* (TR86276|c0_g2_i1 and TR86276|c0_g3_i1) were selected to study the stress tolerance ability under salt and osmotic stress conditions in yeast. The ORF of TR1991|c0_g3_i1, TR29644|c0_g1_i1, TR54730|c0_g1_i1, TR138719|c0_g1_i1, TR86276|c0_g2_i1 and TR86276|c0_g3_i1 were amplified from pGBKT7-*BaAP2/ERF* plasmids of transcriptional activation assay, using primers shown in Table S5, and inserted into the yeast expression vector pYES2 using the in-fusion PCR cloning system. The ORF of two *DREB* genes TR119737|c13_g1_i1 and TR27842|c0_g1_i1 were obtained from pGBKT7-*BaAP2/ERF* plasmids using *Not*I and *EcoR*I restriction enzymes digestion and inserted into the *Not*I and *EcoR*I sites of the yeast expression vector pYES2, which contains a URA3 selection marker driven by the GAL1 promoter. Subsequently, eight pYES2-*BaAP2/ERF* plasmids and the empty pYES2 control plasmids were introduced into yeast strain INVSc1 (Invitrogen, Carlsbad, CA, USA) using a lithium acetate procedure, according to the pYES2 vector kit instructions (Invitrogen, Carlsbad, CA, USA). The transformants were screened by growth on a uracil-deficient synthetic complete (SC-ura) medium with 2% (*w*/*v*) glucose at 30 °C for 2 days.

For the stress assay, yeast cells harboring both pYES2-BaAP2/ERFs and the empty pYES2 vector (control) were incubated in SC-ura liquid medium containing 2% glucose at 30 °C for approximately 20 h with shaking (180 rpm). After incubation, the optical densities of the yeast cell were determined at OD600. The culture samples were adjusted to contain an equal OD600 of 0.4 as a starting concentration in 10 mL of induction SC-ura medium (supplemented with 2% *w*/*v* galactose). After incubation for approximately 24 h, the yeast cell densities were recalculated and adjusted to contain an equal number of cells (OD600 = 2) in 200 μL solutions with 5 M NaCl or 3M Sorbitol for the salt or osmotic stress, and the same quantity of yeast cells was re-suspended in 200 μL of sterile water was served as the control. After incubating at 30 °C for 45 h, the cells were 10-fold serially diluted with sterile water, and 2 μL aliquots of each dilution were spread on SC-ura medium containing 2% (*w*/*v*) glucose and growth performance was compared after growing at 30 °C for 2 days [18,44].

## 5. Conclusions

This is the first report on identification, classification, characterization, and functional evaluation of the *AP2/ERF* gene family in the desiccation tolerant moss *B. argentums*. Eighty-three AP2/ERF predicted proteins were identified from the *B. argenteum* transcriptome and classified within the *AP2/ERF* gene family. The gene expression pattern was analyzed in response to a well characterized dehydration–rehydration based upon RT-qPCR and RNA-seq data. We verified the transactivation activities of 12 representative *BaAP2/ERF* genes. Furthermore, eight *BaAP2/ERF* genes were tested for stress tolerance functions in yeast. We conclude that TR29644|c0_g1_i1 (*DREB*-Ba-unique), TR119737|c13_g1_i1 (*DREB*), TR54730|c0_g1_i1 (*ERF*), TR27842|c0_g1_i1 (*DREB*) and TR86276|c0_g2_i1 (*Soloist*) genes strongly respond to environmental stress and that these genes are correlated with enhanced salt- and osmotic-stress tolerance in transgenic yeast. These genes are promising candidate genes for further functional analysis and demonstrate great potential in plant molecular breeding.

## Supplementary Materials

Supplementary Materials can be found at http://www.mdpi.com/1422-0067/19/11/3637/s1, Figure S1: The 20 most abundant predicted transcription factor families in the *B. argenteum* transcripotome datasets; Figure S2: Phylogenetic analysis of *AP2/ERF* family genes in *B. argenteum*; Figure S3: Phylogenetic analysis of *AP2/ERF* family genes in *B. argenteum* and *P. patens*; Table S1: Sequence alignment results of 25 Ba-clade DREBs using IKP BLAST; Table S2: Primer information of 12 *BaAP2/ERF* genes for RT-qPCR analysis; Table S3: Primer information of 12 *BaAP2/ERF* genes for gene cloning; Table S4: Primer information of 12 *BaAP2/ERF* genes for fusing to pGBKT7 vector; Table S5: Primer information of *BaAP2/ERF* genes for infusing to pYES2 vector.

## Abbreviations

AP2/ERF	APETALA2/Ethylene responsive factor
DREB	dehydration-responsive element-binding protein
ERF	ethylene-responsive factor
RAV	related to ABI3/VP1
RT-qPCR	reverse transcription quantitative real-time polymerase chain reaction
TF	transcription factor
DT	desiccation tolerance
