# Supplementary material for: Identification, Classification, and Functional Analysis of AP2/ERF Family Genes in the Desert Moss Bryum argenteum
Source: Int J Mol Sci. 2018 Nov 19;19(11):3637. doi: 10.3390/ijms19113637 (PMC6275083; doi:10.3390/ijms19113637)
Supplement: Supplementary file 1 [file ijms-19-03637-s001.zip › ijms-376669-supplementary/Figure S 1-S3.pdf]

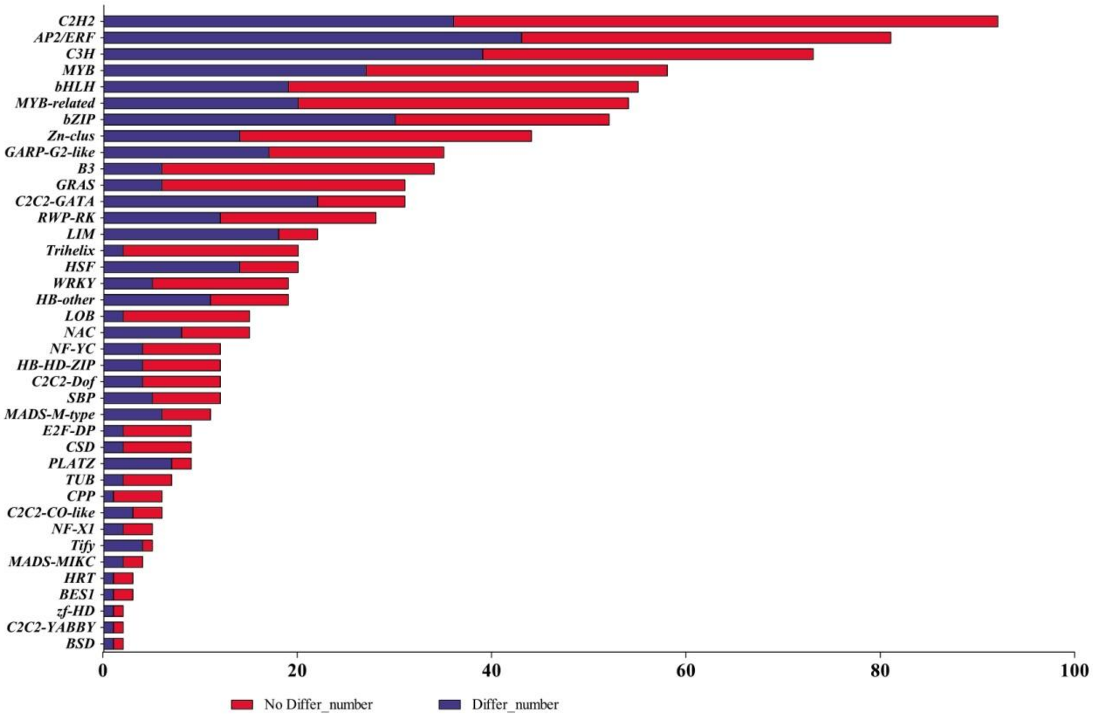

Supplementary Figure 1. The 20 most abundant predicted transcription factor families in the *B. argenteum* transcripotome datasets. The differential expressed TF genes (The blue bars) of the total numbers (the red bars) of each TF family during desiccation and rehydration process were shown.

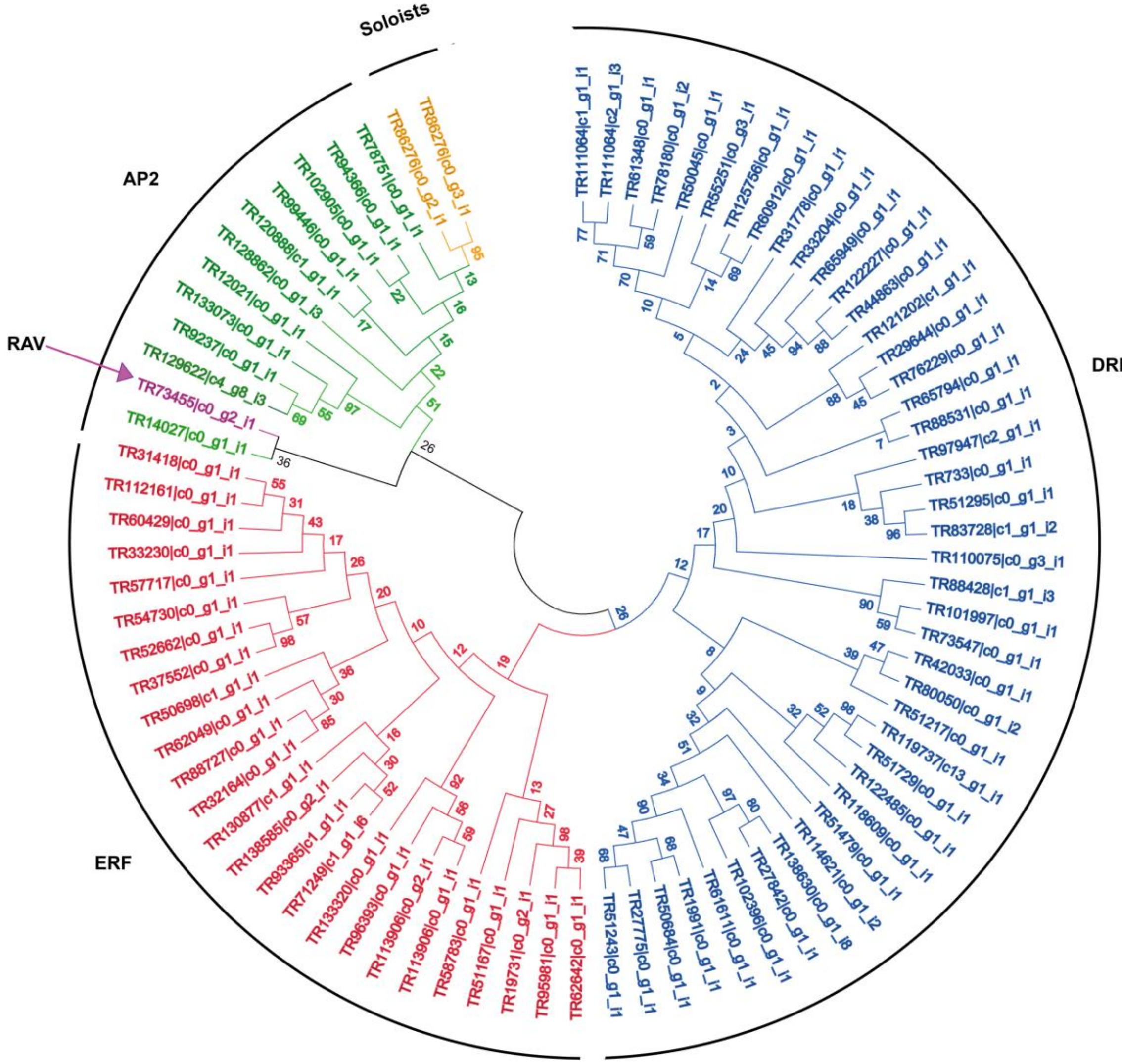

Supplementary Fig. S2. Phylogenetic analysis of *AP2/ERF* family genes in *B. argenteum*. The gene tree was constructed using neighbor-joining method using 83 BaAP2/ERFs, Poisson model with pairwise deletion. Bootstrap values from 1000 replicates were used to assess the robustness of the tree.

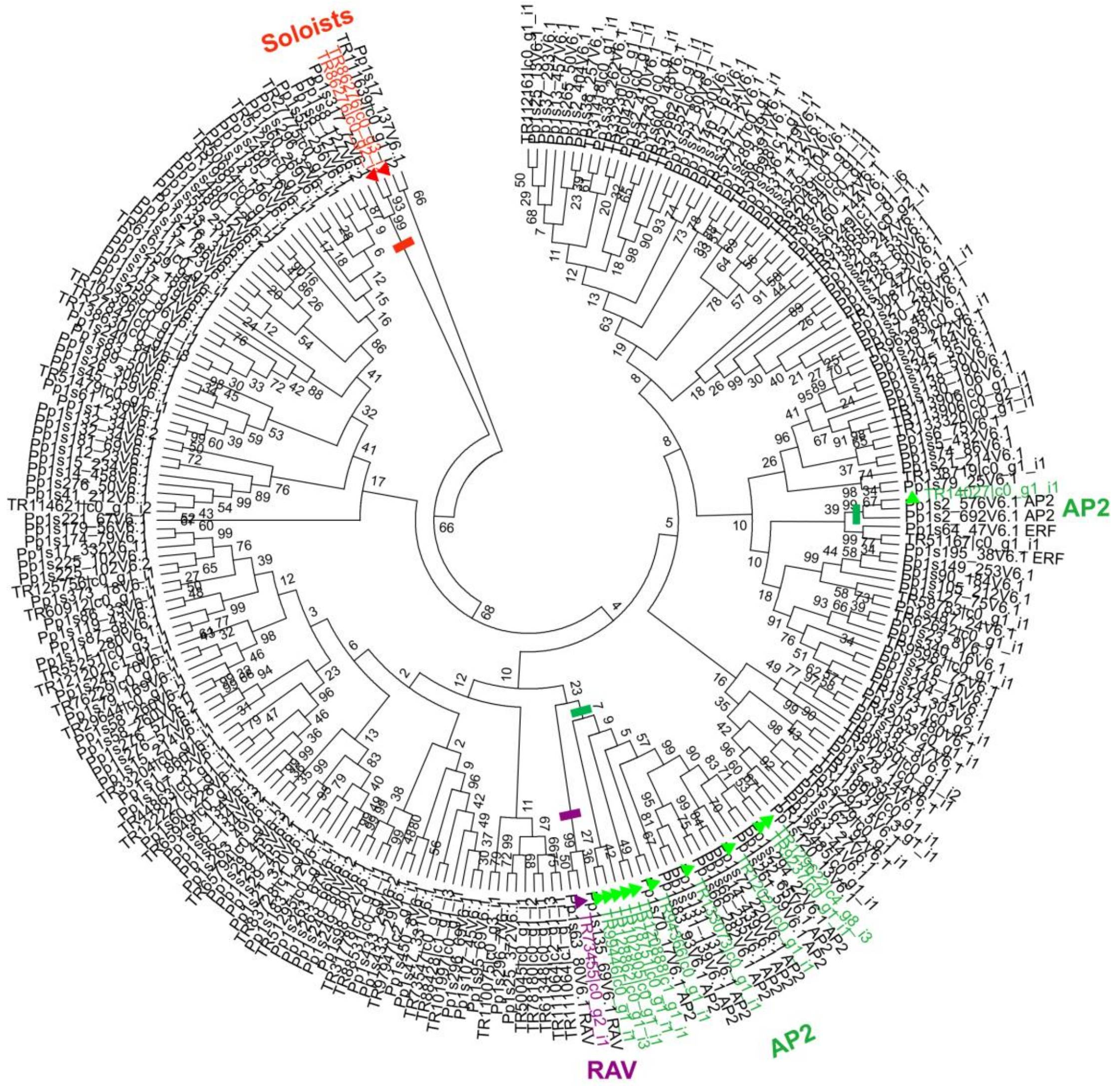

Supplementary Fig. S3. Phylogenetic analysis of *AP2/ERF* family genes in *B. argenteum and P.patens.* Eighty-three BaAP2/ERFs and 171PpAP2/ERFs were used to construct the gene tree. The evolutionary distances were computed using the neighbor-joining method and Poisson model with pairwise deletion, 1000 replicates were used to assess the robustness of the tree.
