# Supplementary material for: Identification, Classification, and Functional Analysis of AP2/ERF Family Genes in the Desert Moss Bryum argenteum
Source: Int J Mol Sci. 2018 Nov 19;19(11):3637. doi: 10.3390/ijms19113637 (PMC6275083; doi:10.3390/ijms19113637)
Supplement: Supplementary file 1 [file ijms-19-03637-s001.zip › ijms-376669-supplementary/Table S1-S5.pdf]

Table S1 **Sequence alignment results of 25 Ba-clade DREBs using IKP BLAST.**

	Gene	Length (aa)	Moss Species	E-value	Identity
1	TR125756_c0_g1_i1	216	*Funaria*	1.59737e-144	200/202(100%)
2	TR60912_c0_g1_i1	279	*Funaria*	2.57357e-121	176/187(95%)
3	TR76229_c0_g1_i1	284	*Funaria*	3.74832e-132	211/227(93%)
4	TR97947_c2_g1_i1	381	*Funaria*	0.0	319/360(89%)
5	TR55251_c0_g3_i1	193	*Funaria*	1.86953e-87	138/156(89%)
6	TR65794_c0_g1_i1	207	*Racomitrium_varium*	1.92769e-105	164/185(89%)
7	TR51295_c0_g1_i1	315	*Funaria*	1.16271e-85	125/143(88%)
8	TR88428_c1_g1_i3	219	*Racomitrium_varium*	5.59893e-71	101/117(87%)
9	TR122227_c0_g1_i1	163	*Funaria*	1.72466e-72	106/123(87%)
10	TR44863_c0_g1_i1	197	*Funaria*	2.08436e-67	107/126(85%)
11	TR29644_c0_g1_i1	273	*Funaria*	2.86133e-126	180/220(82%)
12	TR50045_c0_g1_i1	496	*Funaria*	0.0	277/352(79%)
13	TR65949_c0_g1_i1	326	*Funaria*	1.2845e-180	251/325(78%)
14	TR101997_c0_g1_i1	333	*Racomitrium_varium*	2.19047e-62	97/126(77%)
15	TR126346_c7_g1_i1	649	*Golenkinia_longispicula*	1.85319e-35	57/78(74%)
16	TR121202_c1_g1_i1	235	*Neckera_douglasii*	1.56115e-64	118/181(66%)
17	TR31778_c0_g1_i1	269	*Scouleria_aquatica*	8.1443e-67	104/159(66%)
18	TR83728_c1_g1_i2	380	*Encalypta_streptocarpa*	1.88475e-144	232/376(62%)
19	TR88531_c0_g1_i1	232	*Buxbaumia_aphylla*	2.66208e-32	89/144(61%)
20	TR111064_c1_g1_i1	266	*Neckera_douglasii*	9.94306e-27	60/103(59%)
21	TR61348_c0_g1_i1	312	*Aulacomnium_heterostichum*	6.56064e-37	71/126(57%)
22	TR73547_c0_g1_i1	345	*Pseudotaxiphyllum_elegans*	8.72126e-64	115/205(57%)
23	TR111064_c2_g1_i3	416	*Neckera_douglasii*	5.1885e-22	50/93(54%)
24	TR110075_c0_g3_i1	218	*Racomitrium_varium*	2.30651e-23	55/104(53%)
25	TR78180_c0_g1_i2	260	*Aulacomnium_heterostichum*	8.42958e-27	66/140(48%)

Table S2 **Primer information of 12 *BaAP2/ERF* genes for RT-qPCR analysis.**

Gene	Subfamily	Primer sequence (5'→3')	Length (bp)
TR129622|c4_g8_i3	AP2	GGTCCTGGAACTGTCATCAAC<br>CCAAAGGTGCCAAGGTAGA	230
TR119737|c13_g1_i1	DREB (A-2)	TCTGGACCGGATTGATTTGGA<br>AACGCTGTCGATGCTTTGCA	151
TR27842|c0_g1_i1	DREB (A-5)	CTTCCCAGACTCCATCCCTTCG<br>TTGTCGTCATTTGCGGTTTCC	285
TR1991|c0_g3_i1	DREB (A-5)	CCGACCACGAGGCTTCAAATC<br>CAATACTGGGCGACAATGGCTTA	285
TR42033|c0_g1_i1	DREB (A-6)	GTCGAGGTCTGGGTACGTGAGT<br>CGTGGTGGTGACGGAAGTGA	238
TR125756|c0_g1_i1	DREB unclassified	CATTATAGAACACGGGAAGAAAGC<br>ACGGCACCTTATTCAAATCAAA	272
TR29644|c0_g1_i1	DREB unclassified	CAACATCGTCGCAGAGTCCA<br>CAGGTTCTTCTTGCAGCACAGA	245
TR113906|c0_g1_i1	ERF(B1)	AACGGCACCACCACCCAT<br>TCGTGAGGCTGAGCAAGGA	132
TR54730|c0_g1_i1	ERF(B3)	GGCTGGGCACTTACGACA	126

		CGAGGTTGGCTGAGTAGACG	
TR138719|c0_g1_i1	ERF(B6)	AGAGGCGCAGTCGCAGTCA<br>GCAAGATGCCCAGGTTGGAT	122
TR86276|c0_g2_i1	Soloist	ACACTCCTTTCGTTCTTTACT<br>TGGATGATGCCAATCTTAT	187
TR86276|c0_g3_i1	Soloist	ACTTGGGCACCGTTGATT<br>GCTTCCTCTTCTTACTCAAGATGGA	180

Table S3 **Primer information of 12 *BaAP2/ERF* genes for gene cloning.**

Gene	Primer sequence (5'→3')	Product length(bp)	CDS length(bp)	ORF length(aa)
TR129622|c4_g8_i3	TTGAAGGGGGAGAGTGTTGTT<br>TTTCCACCGAGCTCTCCATCA	818	594	198
TR119737|c13_g1_i1	CTGACATTGCTACAGAGTTCGT<br>AAACCAGCTCCCGGGTCAAT	879	789	263
TR27842|c0_g1_i1	GATTCGGCAGTGAGCAATTGTA<br>TGCATCTCCAACACCAATATG	732	618	206
TR1991|c0_g3_i1	ATGCTTGGGATTTGGGAGCTC<br>GATCACGCTCCAAACAATTGTA	1131	1041	347
TR42033|c0_g1_i1	AGCACGTTTGAAGTTTGTGCG<br>CACCAGAACTGTCAACCAAGAT	1561	1482	494
TR125756|c0_g1_i1	TTGTGTGTGCAATTGAGACCGT<br>TGATTGCTTGAGTGAAGCTTCA	757	648	216
TR29644|c0_g1_i1	ATCGTGTATTTAGACACGGTGT<br>CGGAATGGTTGCTAATGAAACT	924	819	273
TR113906|c0_g1_i1	TTACATGCGGTGGGAGTACTG<br>ACCTAAGCATGATGTTTGCAAG	757	699	233
TR54730|c0_g1_i1	TCGAGTGGAGCTAATTCGATC<br>CAGCATCGTCTGCAAGAACGA	1134	1011	337
TR138719|c0_g1_i1	GTCAGAAGTTCTGTATCTTGCA<br>GTGTCAGCAATCATGTGAAGTA	802	621	207
TR86276|c0_g2_i1	ATCCTACCACGAGCTTCATATC<br>CATAATTCAGTACCATCCACAG	844	759	253
TR86276|c0_g3_i1	ATCCTACCACGAGCTTCATATC<br>GCAGGCTATCATGATAATTCCT	684	612	204

Table S4 **Primer information of 12 *BaAP2/ERF* genes for fusing to pGBKT7 vector. The vector sequence was labeled in red.**

Gene	Primer sequence (5’→3’)
TR129622|c4_g8_i3	CATGGAGGCCGAATTCATGGAGGTGGTGTCGAAATT<br>GCAGGTCGACGGATCCTCATATACGATTTTTGGACAAC
TR119737|c13_g1_i1	CATGGAGGCCGAATTCATGCAGTTGGGAAACAACAG<br>GCAGGTCGACGGATCCTCAATGCTGGTCCGGACC
TR27842|c0_g1_i1	CATGGAGGCCGAATTCATGGTGGACAACGGCAGGAG<br>GCAGGTCGACGGATCCTCAGGAGAAAGACCAGAGCTC
TR1991|c0_g3_i1	CATGGAGGCCGAATTCATGGTTGACAAGCAGAGGA<br>GCAGGTCGACGGATCCTTAAGCTCCGGGGAAGCT
TR42033|c0_g1_i1	CATGGAGGCCGAATTCATGGCAGCAGTGGCACAC<br>GCAGGTCGACGGATCCTCATACACACTGTCTCCAG
TR125756|c0_g1_i1	CATGGAGGCCGAATTCATGAATTTGACAATATCCAGC<br>GCAGGTCGACGGATCCCTAGCTGGACGAGGGCAC
TR29644|c0_g1_i1	CATGGAGGCCGAATTCATGTCATCGGGGAAGTGC<br>GCAGGTCGACGGATCCTCAATAGAGATAGAAAACGTC
TR113906|c0_g1_i1	CATGGAGGCCGAATTCATGGGGTCTCGAGAAGGG<br>GCAGGTCGACGGATCCTCAGAAATATACCTCACAAGA
TR54730|c0_g1_i1	CATGGAGGCCGAATTCATGACCATCATTCCGGGTG<br>GCAGGTCGACGGATCCTCAGAATGGCGGACTTCG
TR138719|c0_g1_i1	CATGGAGGCCGAATTCATGTGCAGGAAGATGAGAG<br>GCAGGTCGACGGATCCCTACGCGGAGGCGGCCGT
TR86276|c0_g2_i1	CATGGAGGCCGAATTCATGGTTAGTATCAGAAAACGG<br>GCAGGTCGACGGATCCTTATGATGACCTCTTAAATGTT
TR86276|c0_g3_i1	CATGGAGGCCGAATTCATGGTTAGTATCAGAAAACGG<br>GCAGGTCGACGGATCCTTATGATGAGCATTTGAGAGC

Table S5 **Primer information of *BaAP2/ERF* genes for infusing to pYES2 vector. The vector sequences were labeled in red.**

Gene	Primer sequence (5’→3’)
TR1991|c0_g3_i1	CAGTGTGCTGGAATTCATGGTTGACAAGCAGAGGA<br>CATGCTCGAGCGGCCGCTTAAGCTCCGGGGAAGCT
TR29644|c0_g1_i1	CAGTGTGCTGGAATTCATGTCATCGGGGAAGTGC<br>CATGCTCGAGCGGCCGCTCAATAGAGATAGAAAACGTC
TR54730|c0_g1_i1	CAGTGTGCTGGAATTCATGACCATCATTCCGGGTG<br>CATGCTCGAGCGGCCGCTCAGAATGGCGGACTTCG
TR138719|c0_g1_i1	CAGTGTGCTGGAATTCATGTGCAGGAAGATGAGAG<br>CATGCTCGAGCGGCCGCCTACGCGGAGGCGGCCGT
TR86276|c0_g2_i1	CAGTGTGCTGGAATTCATGGTTAGTATCAGAAAACGG<br>CATGCTCGAGCGGCCGCTTATGATGACCTCTTAAATGTT
TR86276|c0_g3_i1	CAGTGTGCTGGAATTCATGGTTAGTATCAGAAAACGG<br>CATGCTCGAGCGGCCGCTTATGATGAGCATTTGAGAGC
